# An Explicit Structural Model of Root Hair and Soil Interactions Parameterised by Synchrotron X-ray Computed Tomography

**DOI:** 10.1007/s11538-017-0350-x

**Published:** 2017-10-13

**Authors:** Samuel David Keyes, Konstantinos C. Zygalakis, Tiina Roose

**Affiliations:** 1 Bioengineering Sciences Research Group, Faculty of Engineering and Environment, University of Southampton, Southampton, SO17 1BJ UK; 2 School of Mathematics, University of Edinburgh, Edinburgh, EH8 9YL UK

**Keywords:** Root hairs, Structural modelling, X-ray CT, Rhizosphere, Synchrotron, *In silico*

## Abstract

The rhizosphere is a zone of fundamental importance for understanding the dynamics of nutrient acquisition by plant roots. The canonical difficulty of experimentally investigating the rhizosphere led long ago to the adoption of mathematical models, the most sophisticated of which now incorporate explicit representations of root hairs and rhizosphere soil. Mathematical upscaling regimes, such as homogenisation, offer the possibility of incorporating into larger-scale models the important mechanistic processes occurring at the rhizosphere scale. However, we lack concrete descriptions of all the features required to fully parameterise models at the rhizosphere scale. By combining synchrotron X-ray computed tomography (SRXCT) and a novel root growth assay, we derive a three-dimensional description of rhizosphere soil structure suitable for use in multi-scale modelling frameworks. We describe an approach to mitigate sub-optimal root hair detection via structural root hair growth modelling. The growth model is explicitly parameterised with SRXCT data and simulates three-dimensional root hair ideotypes *in silico*, which are suitable for both ideotypic analysis and parameterisation of 3D geometry in mathematical models. The study considers different hypothetical conditions governing root hair interactions with soil matrices, with their respective effects on hair morphology being compared between idealised and image-derived soil/root geometries. The studies in idealised geometries suggest that packing arrangement of soil affects hair tortuosity more than the particle diameter. Results in field-derived soil suggest that hair access to poorly mobile nutrients is particularly sensitive to the physical interaction between the growing hairs and the phase of the soil in which soil water is present (i.e. the hydrated textural phase). The general trends in fluid-coincident hair length with distance from the root, and their dependence on hair/soil interaction mechanisms, are conserved across Cartesian and cylindrical geometries.

**Electronic supplementary material:**

The online version of this article (doi:10.1007/s11538-017-0350-x) contains supplementary material, which is available to authorized users.

## Background

Root hairs are specialised epidermal cells that can account for more than 90% of a plant’s root system surface area (Bates and Lynch 1996). Their importance for acquisition of poorly mobile plant nutrients such as phosphorus (P) and potassium (K) has been firmly established (Singh Gahoonia et al. 1997). Despite this, the fundamental mechanisms and timescales relevant for transport of P ions at the hair scale are challenging to quantify experimentally (Roose et al. 2016). In natural soils, very little ($<5\%$) of total plant P uptake is accounted for mass flow to the plasma membranes of root and root hair surfaces, and diffusive transport dominates availability (Lambers et al. 2006). Because diffusion coefficients of P ions in field soils are very low, plants rely on sources that are highly localised to root and root hair surfaces (Lewis and Quirk 1967). The length scales of depletion are comparable to the sizes of single soil aggregates (discrete grains of adhered soil materials), making it relevant to consider the spatial dynamics of sorption, desorption and diffusion at substantially sub-millimetre scales (Hinsinger et al. 2005). These premises have long motivated mathematical modelling of P dynamics at the scales of the rhizosphere, the soil region within a few millimetres of the root surface (Barber et al. 1963; Nye 1966; Drew et al. 1969; Barber and Silberbush 1984; Tinker and Nye 2000; Roose et al. 2001; Leitner et al. 2010b; Ptashnyk and Roose 2010; Zygalakis et al. 2011).

The most recent trend in rhizosphere modelling is to use formal upscaling methods, particularly homogenisation, which permit robust upscaling of rhizosphere-scale physical processes into mathematical models at root, plant or even field scales (Roose et al. 2001; Pavliotis and Stuart 2008; Leitner et al. 2010b; Ptashnyk and Roose 2010; Zygalakis et al. 2011). However, such studies almost always represent soil as a homogenous bulk material, or as an idealised array of packed spheres (Roose et al. 2016). In neither of these cases can the resulting models be considered truly mechanistic at the rhizosphere scale (Darrah et al. 2006; Ashley et al. 2006; Carminati and Vetterlein 2012).

Recent studies have used synchrotron X-ray computed tomography (SRXCT) to produce 3D representations of rhizosphere soil morphology, which can be used to parameterise numerical simulations of species transport (Keyes et al. 2013; Daly et al. 2016; Masum et al. 2016). In some instances, homogenisation has been applied to develop upscaled models which rely on explicit numerical solution of micro-scale *cell problems *(using 3D soil geometries meshed from CT data) to derive macroscale equations (Daly et al. 2015; Tracy et al. 2015). However, this successful fusion of SRXCT imaging and mathematical models raises challenges significantly exceeding those encountered when using idealised simplifications (Barber et al. 1963; Nye 1966; Drew et al. 1969; Barber and Silberbush 1984; Tinker and Nye 2000; Roose et al. 2001; Leitner et al. 2010b; Ptashnyk and Roose 2010; Zygalakis et al. 2011).

**Fig. 1:**
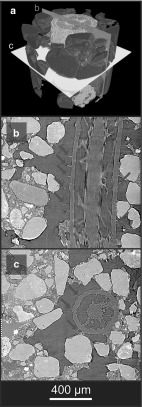
(Color figure online) **a** Three-dimensional SRXCT-derived geometry of a rice root surrounded by rhizosphere soil. Longitudinal (**b**) and transverse (**c**) sections through the grey-level data reveal characteristic features of the rhizosphere: root hairs (red arrows), primary mineral grains (magenta), hydrated textural phase (green) and gaseous pores (yellow)

**Fig. 2:**
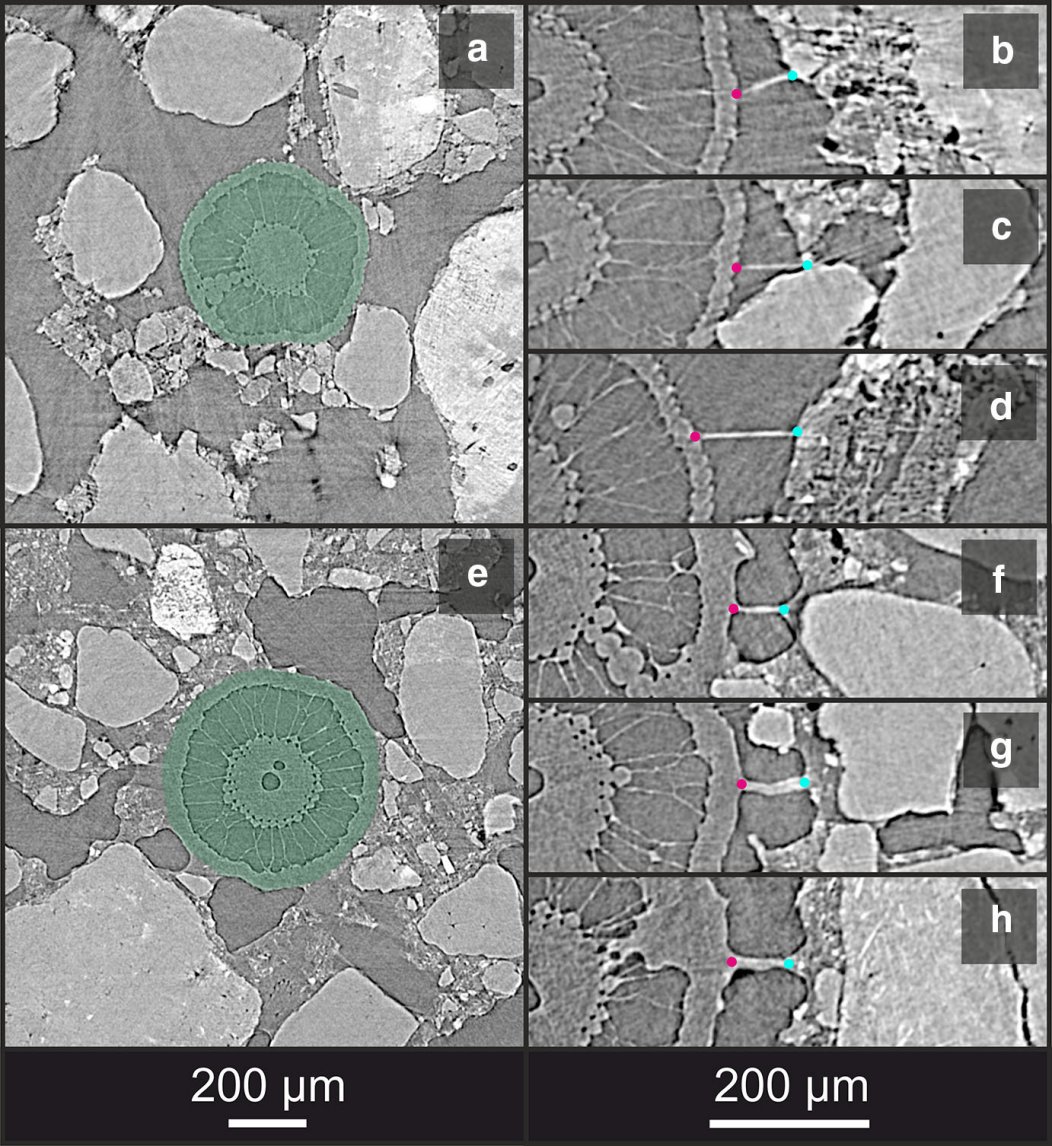
(Color figure online) Representative transverse sections through nodal roots of rice (Oryza sativa cv. DJ123) at both low ($\sim 5\%$ b.v.) water content (**a**–**d**) and high ($\sim 30\%$ b.v.) water content (**e**–**h**), Three representative root hair segments from each root are shown for each case, with the point of initiation at the epidermis (magenta) and the point at which the occluded portion begins (cyan) indicated

A conceptual description of the micro-scale soil structure revealed by SRXCT is shown in Fig. 1 (and Supplementary Figure S2). At this scale, grains of *primary mineral* material (i.e. quartzes and feldspars) are distinguishable from both *gaseous pores* and the *hydrated* *textural phase* in which the primary mineral grains are embedded. The hydrated textural phase comprises the clay, silt and organic fractions, and is the site of the bulk of the soil water at field capacity and below. The primary challenge in using SRXCT descriptions of the rhizosphere to parameterise models and/or understand root hair morphology is the deleterious occlusion of hairs by the non-gaseous soil phases (Keyes et al. 2013; Daly et al. 2016; Koebernick et al. 2017). This is a small-scale instance of the root occlusion problem known from plant-scale imaging, whereby the overlapping X-ray attenuation of roots and water or organic matter phases can render roots indistinguishable in CT data (Heeraman et al. 1997; Kaestner et al. 2006; Lontoc-Roy et al. 2006; Mooney et al. 2012). Figure 2 shows cross sections of nodal roots and representative detail of root hairs for two rice plants (*Oryza sativa* cv. DJ123) imaged at high ($\sim 30\%$ b.v.) and low ($\sim 5\%$ b.v.) water content (Keyes et al. 2013). In each case, individual hair paths are indiscernible once hairs transition to the hydrated textural phase from the gaseous pores. In previous studies, this occlusion has meant that only root hair segments fully within air-filled pore phases can be characterised, which has deleterious implications on the characterisation of the rhizosphere. In a recent study measuring hair length density using SRXCT, this led to underestimation of hair length (Koebernick et al. 2017). In another image-based study explicitly modelling the uptake of P by root hairs, the simulations required that ‘flooding’ of the air-filled pore spaces be virtually imposed, in order to provide a transport continuum for P to travel from soil surfaces to root hairs (Keyes et al. 2013).

In this paper, we extend the scope of future rhizosphere models by developing a mechanistic root hair growth model that explicitly ‘grows’ virtual root hair ideotypes into SRXCT-derived rhizosphere geometries. This enables hypothetical behaviour of hairs in the occluding phases to be studied *in silico* and enables the generation of 3D root ideotypes for future modelling studies. Root hair ideotypes have previously been simulated, but only for a case in which the soil matrix was treated as homogenous, negating the influence of explicit soil structure on hair distributions (Ma et al. 2001; Brown et al. 2012). The fusion of imaging and modelling in this study allows generation of root hair ideotypes which allow hypotheses regarding hair/soil interactions to be explored, and which can be compared using recently developed modelling paradigms (Daly et al. 2016).

## Materials and Methods

The model requires parameterisation using descriptions of 3D, multi-phase rhizosphere geometry derived from SRXCT data. We first describe the imaging methodology and then describe the hair growth model.

### Description of SRXCT Imaging Protocol

Image data were collected using a refinement of the rhizosphere-imaging assay used in (Keyes et al. 2013), standardised for this study using additive manufacturing (3D printing) techniques (Supplementary Figure S1). A plastic seed cup guides nodal roots into soil-filled 1-ml syringe barrels, which are well suited for use as rhizosphere chambers (Keyes et al. 2013; Daly et al. 2016; Koebernick et al. 2017).

#### Growth Media

The growth media was a sand-textured Eutric Cambisol (52.7% sand, 32.8% silt and 14.5% clay) collected from a surface plot at Abergwyngregyn, North Wales, (53014$^\prime$N, 4001$^\prime$W), sieved to $<5\,\hbox {mm}$, air-dried for 2 days at $23\pm 1\,^{\circ }$C and then sieved between 1680 and 1000$\,\upmu \hbox {m}$ to produce a well-aggregated growth medium (the range of diameters of intra-aggregate primary mineral grains extends considerably smaller). Rhizosphere chambers were filled with this prepared medium via gentle tapping, to a bulk density of $\sim 1$ g/ml.

#### Plant Material

Plant material was an upland rice genotype (*Oryza sativa cv. DJ123*). Seeds were germinated for 3 d between moist filter paper (Whatman No 2) in dark conditions at $23\,^{\circ }$C. Following planting to the imaging assays, plants were grown for 14 days in a growth chamber (Fitotron SGR; Weiss Gallenkamp, Loughborough, UK). The rhizosphere chambers were kept hydrated via capillary rise, at a water potential of $\sim -\,0.6$ kPa at the imaging location, until 48 h prior to imaging, during which no water was provided.

#### SRXCT Imaging

Preparation of rhizosphere chambers for SRXCT followed (Keyes et al. 2013; Daly et al. 2016; Koebernick et al. 2017). Imaging was conducted at the TOMCAT beamline of the Swiss Light Source (Paul Scherrer Institute, Villigen, Switzerland). A total of 1601 projections were acquired with an exposure time of 150 ms, over a rotation of $\pi$, at an accelerating voltage of 19 kV. The per-sample scan duration was 4 min, with the projections being reconstructed to 16-bit volume data of dimensions $2560\times 2560\times 2160$ using the Gridrec algorithm (Marone and Stampanoni 2012). Figures 1 and 2 show representative SRXCT volume data gathered using the growth/imaging protocol.

### Structural Simulation of Root Hair Growth

At the scale of the whole root system architecture, functional–structural models have an established role in testing and generating hypotheses (Diggle 1988; Lynch et al. 1997; Pagès et al. 2004; Leitner et al. 2010a; Dunbabin et al. 2013; Pagès and Picon-Cochard 2014). The functional–structural root hair model described here uses SRXCT-derived rhizosphere geometry to explicitly parameterise 3D hair ideotypes. The approach is similar to the time-stepped, tip-extending approaches common to root growth modelling (Leitner et al. 2010a), but incorporates a collision-avoidance routine that allows explicit soil structure to influence growth orientation.

Two modes of operation are envisioned: the virtual completion of partially visible root hair segments (such as the hairs shown in Fig. 2) and the growth of entirely synthetic hairs, using randomised initiation points sampled on a root surface. The parameterisation requirements have been constrained to a minimal list, conducive to determination using literature-derived values and/or data from simple experiments:

1. A three-dimensional representation (in regular, 3D array form) of the rhizosphere domain, in which primary mineral, hydrated textural, gaseous pore and root/root hair phases are denoted by discrete values.
2. The coordinates of the start point for modelling of growth of each hair and a vector describing the initial growth heading.
3. The total length ($l_{\mathrm{tot},i}$) to which each of the *i* hairs should grow.

### Assumptions

**Fig. 3:**
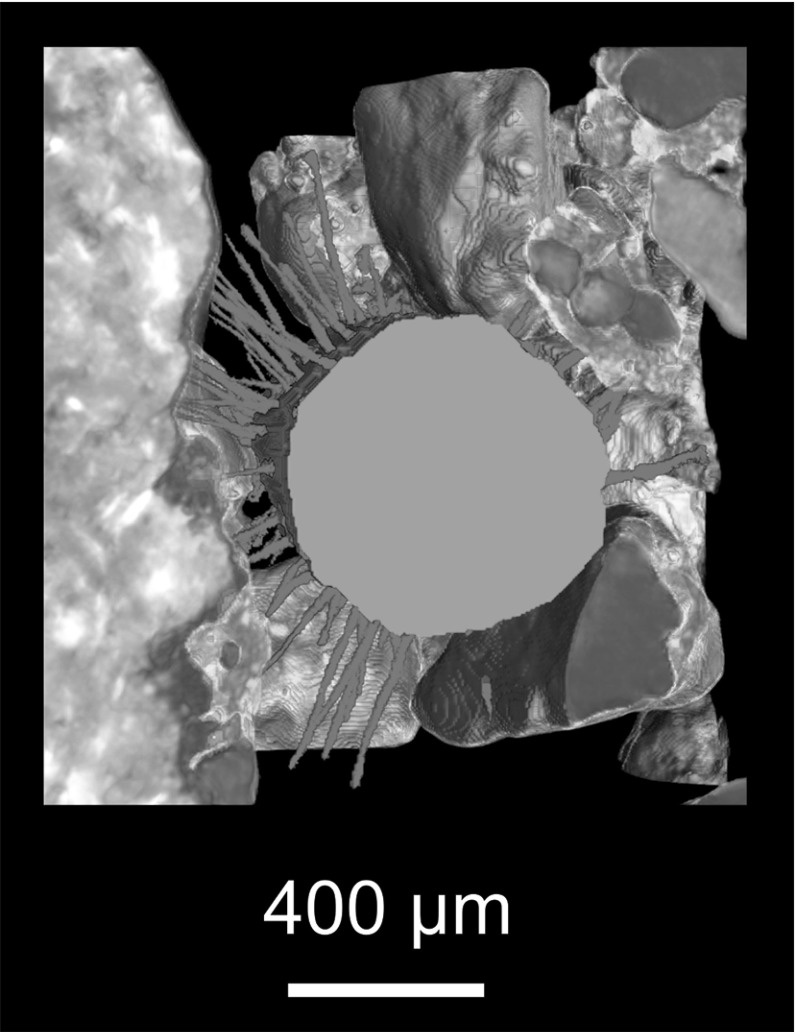
(Color figure online) 3D rendering of segmented SRXCT data, showing a nodal root of rice (Oryza sativa cv. DJ123) in green, with hairs paths in the gaseous pore phase shown in red. Hairs display low tortuosity in the gaseous pore phase

To contrain the behaviour of the model, the following assumptions were made regarding hair growth dynamics:

(**A1**): Unimpeded hairs grow with low angular deviation (Sieberer et al. 2005; Grierson et al. 2014) . This concurs with observations in gel media (Gilroy and Jones 2000) and SRXCT data (Fig. 3).
(**A2**): Hairs cannot ingress into primary mineral grains. These hard, weathering-resistant minerals (primarily quartz in this study) are assumed to be resistant to penetration via mechanical processes and to dissolution via exudation of organic acids (on any timescales close to that of the hair lifecycle).
(**A3**): Hairs can ingress into the hydrated textural phase, suggesting hairs can freely enter the hydrated textural phase (Figs. 2, 3).
(**A4**): Hair growth orientation is not influenced by the sensing of endogenous chemical species at the root tip. Though nodulation factors can initiate hair curling (Esseling and Emons 2004), their incidence is not identifiable, and such effects are neglected.
(**A5**): Thin fluid films are assumed to exist on the surfaces of primary mineral grains (Tuller and Or 2005).

### Parameterisation Protocols

This section details the means used to parameterise the cases described in the study. At points, additional functionality is mentioned that is of relevance to potential future uses of the model.

#### Collision Geometry

The model uses explicit 3D descriptions of gaseous pore, primary mineral, hydrated textural and root phases to constrain the mechanics of hair growth. Discrete definition of these 3D domains using SRXCT data requires solving the *segmentation problem*, a well-known challenge in X-ray CT of plant and soil systems (Ketcham and Carlson 2001; Schlüter et al. 2014; Roose et al. 2016). In many cases, global histogram approaches which separate regions using discrete grey-level ranges (such as Otsu’s method) fail to discriminate clearly between phases (Otsu 1979; Vogel et al. 2010; Houston et al. 2013). Even at synchrotron resolution ($\sim 1\,\upmu \hbox {m}$), the major components of the hydrated textural phase (i.e. clays, metal sesquioxides and humic particles) have characteristic particle sizes ($<2\,\upmu \hbox {m}$) largely below the threshold of detectability (Houston et al. 2013).

**Fig. 4:**
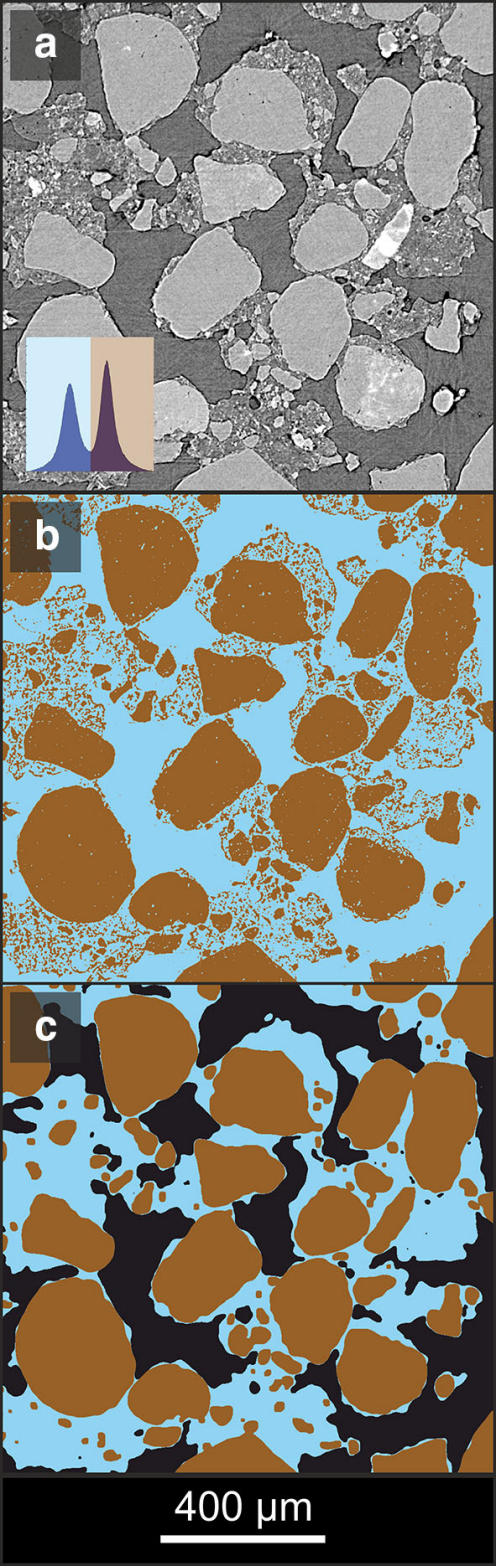
(Color figure online) A comparison of soil segmentation achieved using a global thresholding method (Otsu’s method) and a heuristic method (WEKA). In the raw data histogram (**a**), two peaks encode the three qualitatively distinct phases (primary mineral grains, hydrated textural phase, and gaseous pores). Global segmentation (Otsu) results in erroneous co-classification of gaseous pore and hydrated textural phases from the lower peak, and of primary minerals and hydrated textural phases from the upper peak (**b**). Using two passes of the heuristic method, a three-phase model is produced (**c**), where gaseous pore, primary mineral and hydrated textural phases are discretely represented

A recent review of prevalent segmentation approaches for $\upmu$CT data of porous materials (Iassonov et al. 2009) found that the lowest nominal-to-actual segmentation error and highest noise tolerance were achieved using a heuristic approach (Berthod et al. 1996). We use a fast random forest classifier (‘trainable WEKA segmentation’) implemented in FIJI (Schindelin et al. 2012; Arganda-Carreras et al. 2017) for segmentation (full technical details of which are given in Supplementary Materials). The classifier used entropy, neighbours, variance, and Hessian image measures, at $\sigma =2$ to $\sigma =16$, and was iteratively trained on 10 sub-sampled slices of each SRXCT dataset (Koebernick et al. 2017). A two pass approach was used, using an initial training and classification step to differentiate pore and non-pore phases, then masking pore voxels from the grey-level data, training a second time, and applying the classifier to differentiate between primary mineral and hydrated textural phases. Figure 4 shows a comparison of results between a common global threshold approach (Otsu’s method) and the heuristic approach. The primary mineral phase was next filtered by volume to remove small particles with an effective diameter smaller than the model step length ($32\,\upmu \hbox {m}$). The significance of this step is described later.

#### Hair Initiation and Heading

**Fig. 5:**
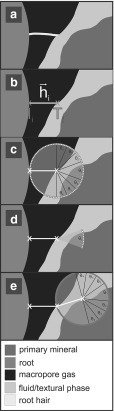
(Color figure online) Schematic representation of a single growth step. The growth model approximates the visible segment of a partially visible hair (**a**) using a vector $\vec {h}_I$ (**b**). The test vectors (end-points shown in white) are classified by angle (**c**), with disallowed locations ($\theta >\frac{\pi }{2}$ and/or $p_\mathrm{test} \in \mathbf{P}$) indicated in red. The set of lowest angle containing at least one allowable test vector is selected (**d**). A randomly selecting a vector from this set (from two possibilities in this instance) becomes the growth vector, which in turn becomes the initiation vector ($\vec {h}_I$) for the next iteration (**e**)

The model requires a start point for each hair and an initial heading. If existing partial hair paths are available (i.e. from SRXCT), a set of all partially visible hair segments ($\mathbf{H}_{{\varvec{p}}}$) is defined, each of the *i*hairs being represented by a vector approximation $\vec {h}_i$ between the ‘initiation point’ ($I_i$) and the ‘transition point’ ($T_i$) (Fig. 5), the transition point being the point at which ‘synthetic’ growth begins. Experimental observation reveals the tortuosity of unconstrained hairs within gaseous pore space to be very low (Figs. 2, 3), (**A1**), such that the Euclidian length of vector $\vec {h}_i$ (denoted $l_\mathrm{r}$) represents a good approximation to the actual length of a visible hair segment. According to assumption **A3**, the vector $\vec {h}_i$ also defines the initial virtual growth direction of hair *i*in the model.

If entirely synthetic hairs are grown, the transition points ($T_i$) can sampled from the root surface geometry, using the SRXCT images as parameterising data. The initial heading vector ($\vec {h}_i$) of each hair is defined as the unit normal to the root surface at the point ($T_i$), pointing into the soil domain.

#### Hair Length

The model requires a length to which each hair should be synthetically grown. The procedure differs depending on whether existing hair paths are to be completed, or whether entirely synthetic hairs are grown. It also varies depending on whether a fixed length or a length distribution is used to define the lengths of hairs in the grown population.

Where partial hairs (visible length $l_{\mathrm{r},i}$) are to be completed, the true total length ($l_{\mathrm{tot},i}$) of each hair is unknown. Thus a value for $l_{\mathrm{tot},i}$ must be imposed such that a virtual growth length ($l_{\mathrm{v},i}$) can be computed according to the relationship ($l_{\mathrm{v},i} =l_{\mathrm{tot},i} -l_{\mathrm{r},i}$). When fully synthetic hairs are grown, the growth length is simply the total length ($l_{\mathrm{v},i} =l_{\mathrm{tot},i}$). The simplest solution to the assignment of $l_{\mathrm{tot},i}$ is to assign a constant length ($l_{\mathrm{tot},i} =c$) to all partially visible hair segments (set $\mathbf{H}_{{\varvec{p}}}$), where *c* can be either an ideotypic value, or an experimental value from the literature.

Experimental results suggest that real root hairs may follow a unimodal normal-type distribution (see Supplementary Figure S5). A Weibull distribution can be used to model such data, being applicable a wide range of potential distributions (including the normal distribution). The parameterised function (*W*(*l*)) can then be sampled for virtual growth lengths ($l_{\mathrm{v},i}$) using survival analysis (Kleinbaum and Klein 2010). This allows a value of $l_{\mathrm{v},i}$ to be estimated for each $l_{\mathrm{r},i}$ for a set of partially visible hairs, such that the distribution of $l_{\mathrm{tot},i}$ matches the Weibull distribution fitted to experimental data.

Briefly, a Weibull distribution with parameters $({\alpha ,\beta }$) becomes the probability distribution of the root hair length, denoted by $f({x,\alpha ,\beta }$). A partially visible root hair is taken to have ‘survived’ up to length $l_{\mathrm{r},i}$. For the rest of its length $l_{\mathrm{v},i}$ (called the *future lifetime*), a probability distribution function given by:

1 $$\begin{aligned} p_i \left( l \right) =\frac{f\left( {l+l_{\mathrm{r},i} ,\alpha ,\beta } \right) }{1-g\left( {l_{\mathrm{r},i} ,\alpha ,\beta } \right) }, \end{aligned}$$

where $g( {l_{\mathrm{r},i} ,\alpha ,\beta })$ is the cumulative distribution function of the Weibull distribution satisfying:

2 $$\begin{aligned} g\left( {x,\alpha ,\beta } \right) =1-\mathrm{e}^{-\left( {\frac{x}{\beta }} \right) ^{\alpha }}. \end{aligned}$$

To sample the growth length ($l_{\mathrm{v},i}$) of a root hair with visible length $l_{\mathrm{r},i}$, Eq. 1 is sampled using the standard inversion formula for one-dimensional probability distributions. An example of the use of this procedure is given in Supplementary Materials.

### Growth Model Description

Hairs elongate by adding tip material in a stepwise manner, with the number of growth steps ($m_i$) depending on the virtual length ($l_{\mathrm{v},i}$). To avoid hairs traversing primary mineral grains, the step length is set to be smaller than the diameter of the smallest primary mineral grains in the soil matrix morphology. The step number for each hair is then given by $m_i =\frac{l_\mathrm{v} }{r}$. A sensitivity analysis of the relationship between *r*and the hair morphology can be found in Supplementary Materials. This step defined the particle filtering threshold ($\varnothing >32\,\upmu \hbox {m}$) described above.

The stages of the growth model are now outlined (Fig. 5):

**Stage 1** A sphere of radius *r*is centred at the end-point of the vector $\vec {h}_i$ (i.e. the *seed point*, $p_\mathrm{seed}$), which for the initial growth step is the transition point ($T_i$) (Fig. 5c). A user-defined number (*n*) of quasi-equally spaced *test points* ($p_\mathrm{test}$) is placed on the surface of this sphere using a spiral approximation (Kuijlaars and Saff 1997). A *test vector* ($\vec {h}_{\mathrm{test},n}$, the vector between $p_\mathrm{seed}$ and $p_{\mathrm{test},n}$) is then defined for every test point. Hair growth proceeds by randomly choosing a test vector which satisfies the criteria detailed below.

**Stage 2** All test points are first classified according to the angle ($$I_{i,n}$$) formed between $$\vec {h}_i$$ and $$\vec {h}_{\mathrm{test},n}$$. Each angle $$I_{i,n} $$ is computed according to:

3 $$\begin{aligned} I_{i,n} =\left| {2\hbox {arctan}\left( {\frac{\sqrt{x^{2}+y^{2}}-x}{y}} \right) } \right| , \end{aligned}$$

where $$x_n =\Vert \vec {h}_i \times \vec {h}_{\mathrm{test},n}\Vert $$, and $$y_n =\vec {h}_i\vec {h}_{\mathrm{test},n}$$.

**Stage 3** The set of test vectors is sorted on the magnitude of ($I_{i,n}$) into *k*non-overlapping, user-defined sets (${\varvec{\theta }_{1} ,\varvec{\theta } _{2} ,\varvec{\theta }_{3} \ldots \varvec{\theta }_{\varvec{k}} }$), each of which denotes a range of angles, and has a width ($\frac{\pi }{2k}$). This is shown graphically for a 2D case (where $k=4$) in Fig. 5c. The upper bound of set $\varvec{\theta }_{\varvec{k}}$ (and thus the maximum growth deviation between steps) is $\pi /2$ deviation from the previous direction ($\vec {h}_i$).

**Stage 4** Three validity tests are then applied to all test vectors: (a) whether the end-point is within the rhizosphere domain, (b) whether the angle is in range ($I_{i,n} <( {\frac{\pi }{2}})$), (c) whether the end-point enters any primary mineral grains ($p_\mathrm{test} \in \mathbf{P}$). Invalid vectors are removed.

**Stage 5** To introduce a semi-stochastic nature to hair growth, vectors are randomly selected from set ($\varvec{\theta }_{\varvec{j}}$), where *j* is the lowest value in the range ($j\in \mathbb {Z}|1\le j\le k$) for which ($\varvec{\theta }_{\varvec{j}}$) is non-empty. If no point can be found satisfying ($p_{\mathrm{test},n} \in \varvec{\theta }_{1} \ldots \varvec{\theta }_{\varvec{k}}$), then growth of the hair ceases. The selected growth vector becomes the initiation vector ($\vec {h}$) for the subsequent growth step.

Sensitivity analyses to confirm correlation of experimental, sampling and grown hair length distributions, and to assess the influence of step length, are detailed in Supplementary Materials.

For clarity in the ensuing descriptions, the term ‘biological replicate’ ($R_\mathrm{b}$) refers to a separate plant sample imaged using SRXCT, and ‘computational replicate’ ($R_\mathrm{c}$) refers to a unique set of hairs generated using the model.

### Hair Growth Conditions

**Fig. 6:**
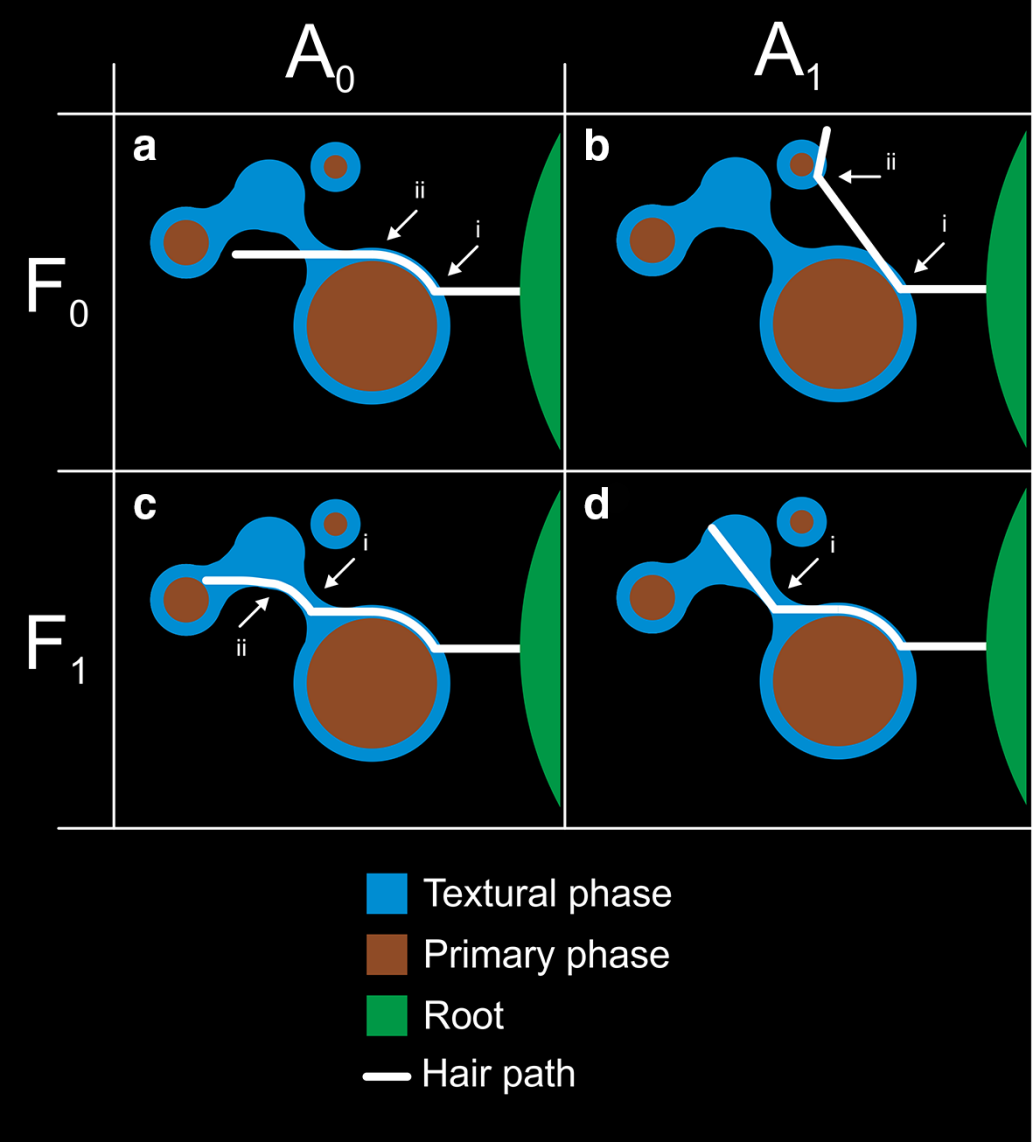
(Color figure online) Conceptual diagram of the four hypothetical growth conditions. Under $\hbox {A}_{0}\hbox {F}_{0 }$ (**a**), hairs always attempt to elongate in the direction of their initial heading. At point (a$_{i}$), the hair deviates to bypass a grain, but continues along its initial heading when unimpeded (a$_{ii}$). Under $\hbox {A}_{1}\hbox {F}_{0 }$ (**b**), hairs continue along their most recent heading. At point (b$_{i}$), the hair deviates upon contact with the particle, continuing along this new heading and deviating a second time at (b$_{ii}$). Under A$_{0}$F$_{1 }$ (**c**), the hydrated textural phase boundary constrains the heading at (c$_{i}$), but the hair elongates in the direction of its initial heading when unimpeded (c$_{ii}$). Under A$_{1}$F$_{1}$ (**d**), the hair is constrained by the fluid film on the surface of the first grain, deviates upon contact with the hydrated textural phase boundary at (d$_{i}$) and then elongates freely along a different heading to the initial direction of growth

Putative hair–soil interaction mechanisms can be simulated by varying the control parameters of the model. The following conditions are considered, shown schematically in Fig. 6.

#### Condition A$_{0}$: Constant Initiation Vector

4 $$\begin{aligned} (\vec {h}_{i,m}=\vec {h}_{i,0}) \end{aligned}$$

The growth angle $I_{i,n}$ at a given step (*m*) is computed relative to the original initiation vector $(\vec {h}_{i,0}$) rather than the preceding growth vector ($\vec {h}_{i,m-1}$). Hair tortuosity is thus limited to the minimal deviation required to avoid primary mineral phases, with growth direction following the original heading wherever possible (Fig. 6a). This approximates the case where hair rigidity has a limiting effect on tortuosity.

#### Condition A$_{1}$: Changing Initiation Vector

5 $$\begin{aligned} (\vec {h}_{i,m}=\vec {h}_{i,m-1}) \end{aligned}$$

The growth angle $I_{i,n}$ at a given step (*m*) is computed relative to the previous growth vector $(\vec {h}_{i,m-1})$. Sufficient interactions with primary mineral grains can thus allow hairs to become oriented at an angle of greater than $\pi /2$ to the original growth vector $(\vec {h}_{i,1})$, i.e. back towards the root surface (Fig. 6b). This approximates the condition where hair tortuosity is dominated by interactions with the soil geometry.

#### Condition F$_{0}$: Hairs Free to Enter/Exit Fluid

Hairs are permitted to freely cross boundaries between fluid and gaseous pore phases (Fig. 6c).

#### Condition F$_{1}$: Hairs in Fluid Confined to Fluid

6 $$\begin{aligned} \left( p_\mathrm{seed} \in {\varvec{S}}\rightarrow p_\mathrm{test} \in {\varvec{S}} \right) \end{aligned}$$

Hairs may enter the hydrated textural phase (**S**), but remain constrained to growth within it. This approximates the case where the free surface energy at the gaseous pore/fluid interfaces constrain hair growth direction, meaning the hair must deviate according to the morphology of these interfaces (Fig. 6c). Experimental data qualitatively indicate that hairs can freely enter the hydrated textural phase (Fig. 2), but hairs exiting it are rarely observed.

#### Combinations of Cases

Models were run using all permutations of the above conditions (ie $\hbox {A}_{0}\hbox {F}_{0},\, \hbox {A}_{0}\hbox {F}_{1},\, \hbox {A}_{1}\hbox {F}_{0},\, \hbox {A}_{1}\hbox {F}_{1}$), to compare their relative influences on hair morphology. Control cases were run in which explicit soil phases were neglected, approximating a homogenous soil condition (Ma et al. 2001; Leitner et al. 2010b).

### Rhizosphere Geometries

**Fig. 7:**
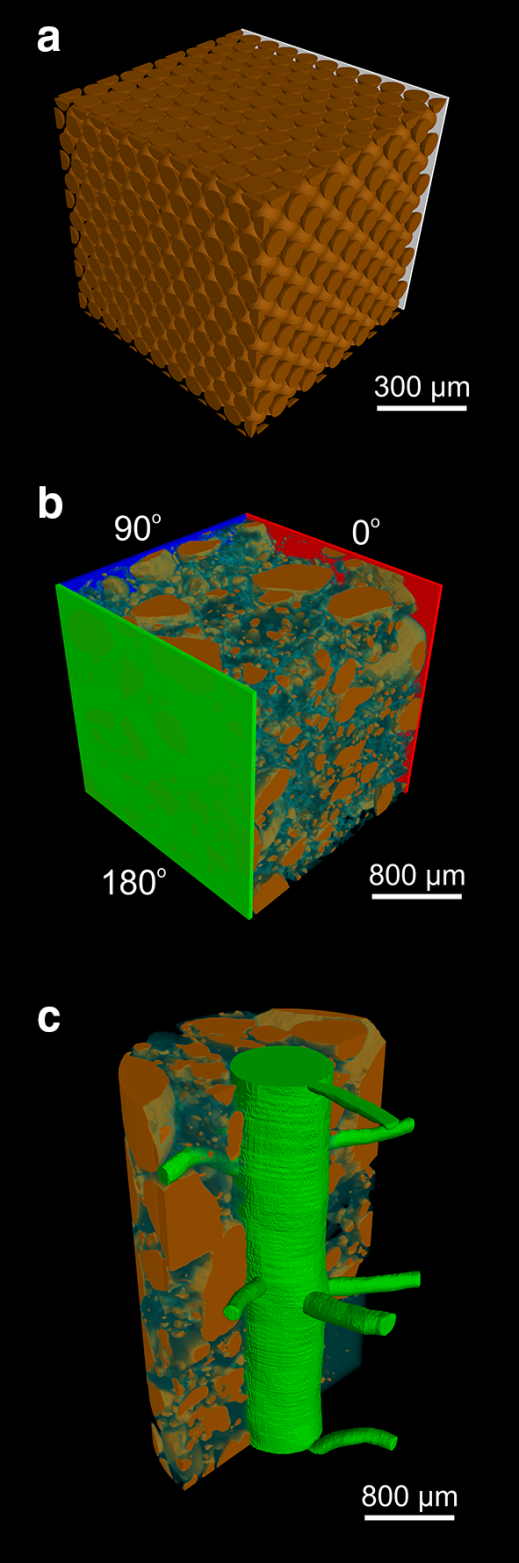
(Color figure online) Schematic of different geometries for parameterising hair growth paths. **a** Synthetic Cartesian soil geometry with uniform spheres ($90\,\upmu \hbox {m}$ HCP shown). **b** The SRXCT-derived Cartesian geometry, in which computational replicates of virtual hair growth were seeded normal to the $0^{\circ }$, $90^{\circ }$ and $180^{\circ }$ faces of the cube (represented by the red, blue and green planes). **c** A cylindrical SRXCT-derived hair geometry, in which computational replicates of virtual hair growth were seeded normal to the root surface

A suite of idealised and SRXCT-derived soil geometries was defined to assess hair morphology under different grain sizes and packing arrangements (Fig. 5). In previous mathematical models that have used explicit 3D geometry, root surfaces have often been idealised as flat (hereafter referred to as *Cartesian*), rather than curved (hereafter referred to *cylindrical*) planes, both shown in Fig. 7 (Ptashnyk and Roose 2010; Zygalakis et al. 2011). Here we consider Cartesian geometries defined using both idealised spheres and SRXCT data, in which hairs initiate from a single face of the cubic domain. Cylindrical geometries are also defined, using SRXCT data, in which hairs initiate from the surface of a segmented root.

#### Idealised Cartesian Soil Matrices

Cubic domains containing either body-centred cubic (BBC) or hexagonal close-packed (HCP) arrays of idealised (spherical) soil particles were generated using FIJI (Fig. 7a). Two sphere diameters were used: the mean inter-hair spacing measured on the root surface ($90\,\upmu \hbox {m}$), and a value characteristic of the lower range of primary mineral grain diameters in the SRXCT data ($270\,\upmu \hbox {m}$). The two geometries represent the body-centred cubic packing (BCC) used in a previous modelling study (Zygalakis et al. 2011) and a hexagonal close-packed (HCP) configuration, the highest theoretically achievable sphere packing density (Fig. 9).

#### SRXCT-Derived Cartesian Soil Matrices

A cubic domain was randomly selected from SRXCT data of a control (i.e. no root) Eutric Cambisol sample (Fig. 7b). The cube dimensions ($1.86 \times 1.86 \times 1.86\,\hbox {mm}$) were defined such that the area of one face was equal to the mean area of the root surfaces in the cylindrical geometries. The volume fractions within this cube were: primary mineral = 32.5%, hydrated textural = 32.5%, gaseous pores = 35%. The model was used to grow hairs from 0$^{\circ }$, 90$^{\circ }$ and 180$^{\circ }$ faces of the cube, using ten computational replicates of every condition.

### Cylindrical Growth Model Scenarios

To produce the cylindrical rhizosphere domains, three biological replicates were randomly selected from the rice root SRXCT data (Fig. 7c). A distance of soil extending to $\sim 600\,\upmu \hbox {m}$ from the root was defined, encompassing the maximal root hair length.

**Table:** 

	Gaseous pore (%)	Hydrated textural (%)	Primary mineral (%)
R1	23.7	37.5	38.8
R2	35.5	24.6	39.9
R3	42.6	15.3	42.1

#### Hair Parameters

Hairs paths are fully synthetic in each case, being seeded from randomly distributed transition points on the root/cube surface. To enable more straightforward comparison, the hair length was set to $l_{\mathrm{tot},i} =500\,\upmu \hbox {m}$ in all cases. Examples of the survival analysis approach are documented in Supplementary Materials. Since rice hairs are $<500\,\upmu \hbox {m}$, the value of hair length was chosen to encompass the entire range of experimentally observed values (Nestler et al. 2016).

Hair density can be defined in a number of ways, using literature, experimental or ideotypic values. In the present case, we used SRXCT data, using FIJI to manually count hair initiations on the root surface of a randomly selected biological replicate, grown and imaged under the conditions described above. Counting fully and partially visible hairs over 3 mm of root length yielded a value of 210 hairs mm$^{-1}$. Using the mean diameter of the root ($600\,\upmu \hbox {m}$) to convert to a value per unit area yielded 121 hairs mm$^{-2}$. This value was used in all models to allow direct comparison.

#### Hair Morphology Quantification

**Fig. 8:**
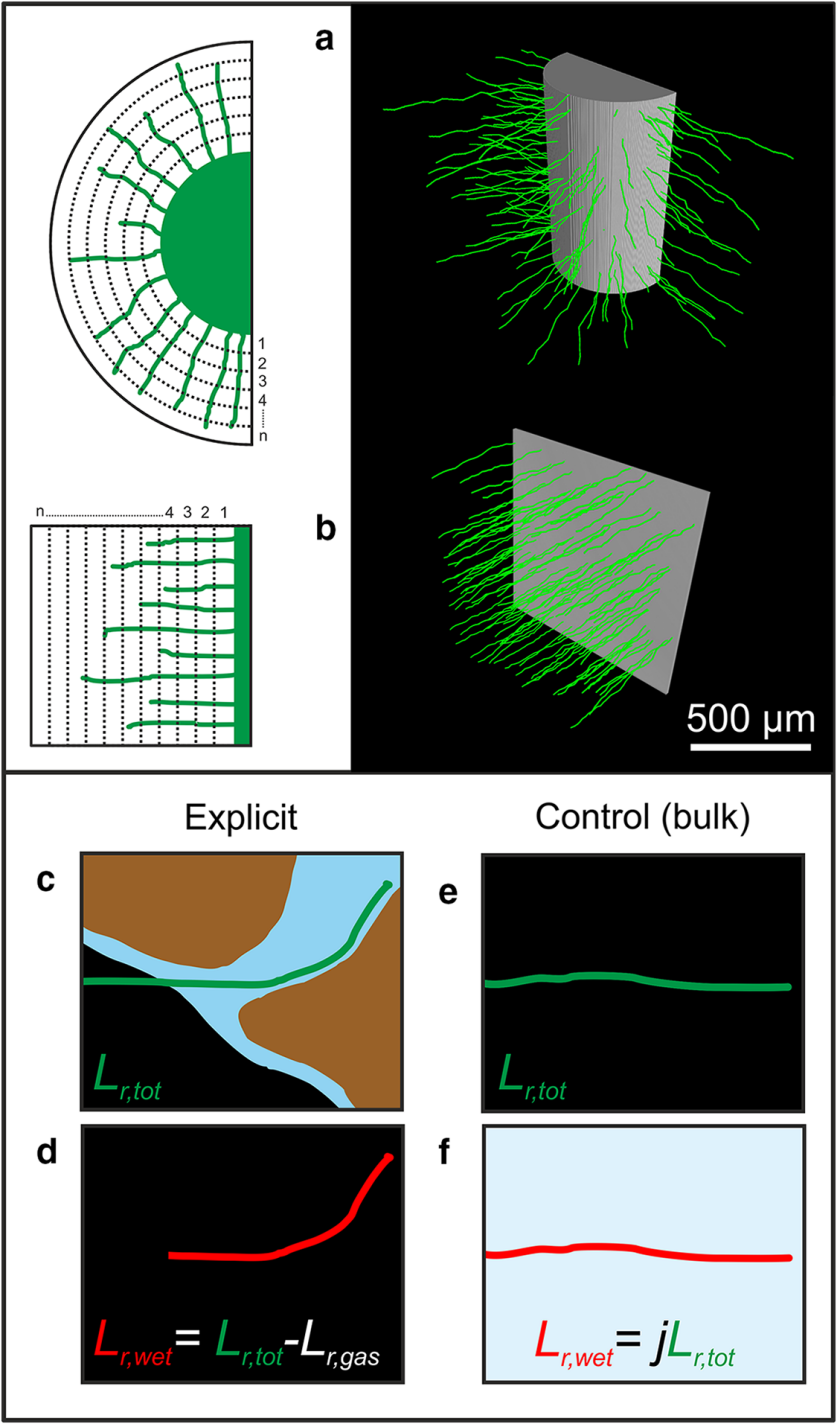
(Color figure online) Schematic illustrating the geometry used for root hair length quantification with distance from the initiating surface. For both cylindrical (**a**) and Cartesian (**b**) geometries, the hair length ($L_{\mathrm{r},\mathrm{tot}}$) enclosed within each of *n* annular sub-volumes is recorded. The fluid-coincident hair length ($L_{\mathrm{r},\mathrm{wet}}$) under explicit growth conditions (**c**) is calculated by neglecting the gaseous pore-coincident fraction of $L_{\mathrm{r},\mathrm{tot}}$ (**d**). For the homogenous controls in which fluid is not explicitly considered (**e**), an approximation of the wetted length is given by multiplying $L_{\mathrm{r},\mathrm{tot}}$ by the volume fraction of hydrated textural phase (*j*) in the non-primary mineral domain (**f**)

The measure of length density ($L_\mathrm{v}$) is common to root system analysis (Pierret et al. 2000). It was modified in this case to a simple measure of length integrated over distance, removing the $r^{2 }$ factor in volume with radial distance (*r*) in the cylindrical geometries to allow direct comparison of Cartesian and cylindrical cases. Root hair length was quantified for increasing distance from the initiating surface, integrating over a set distance of $50\,\upmu \hbox {m}$ in each case (Fig. 8a, b). Two measurements were defined (Fig. 8c–f): the root length with radial distance ($L_{\mathrm{r},\mathrm{tot}}$) and the fluid-coincident root length with radial distance ($L_{\mathrm{r},\mathrm{wet}}$). Because root hairs uptake micro-nutrients from the fluid phase, the proportion of hair surface area in direct fluid contact is an important factor. Comparing fluid-coincident densities between hair morphologies thus gives an approximation as to the difference in P uptake potential between hair ideotypes. The short length scales of P diffusion in soil also mean that near the root, there may be competition between the root surface and hairs for the same P sources (Keyes et al. 2013). Thus the distribution of hair density with distance from the root is also of interest.

**Fig. 9:**
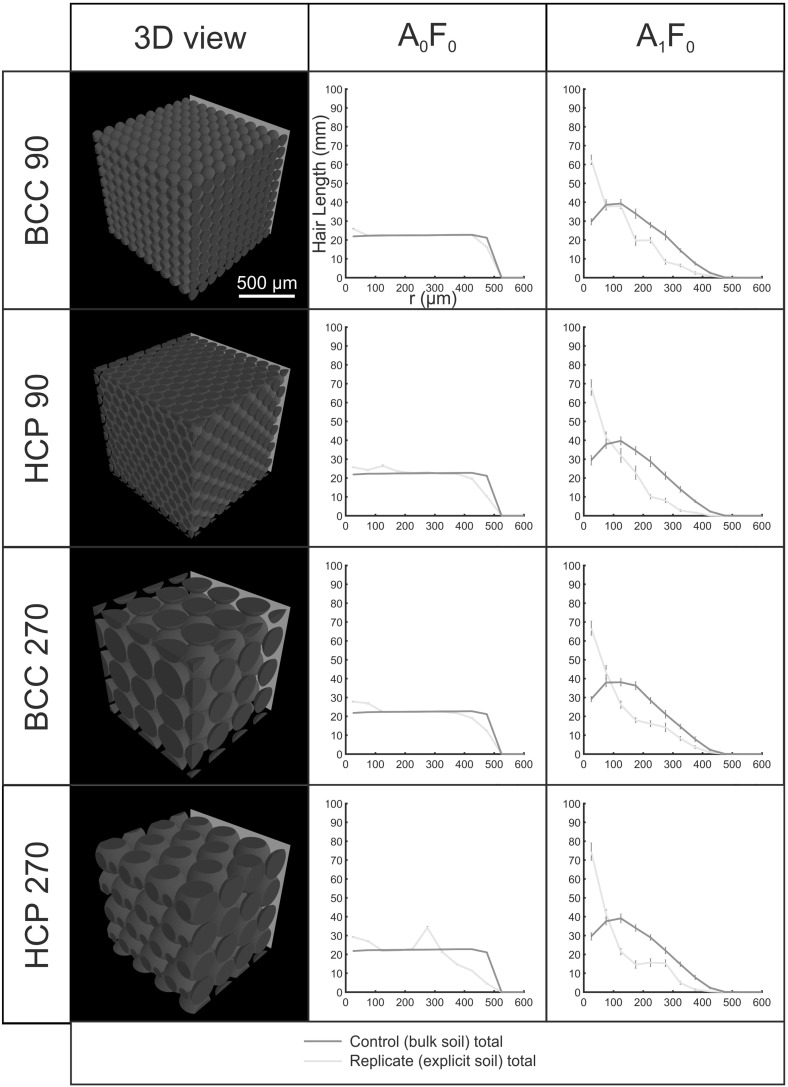
(Color figure online) Hair length profiles with distance (*r*) from the initiating surface for $90\,\upmu \hbox {m}$ BCC, $90\,\upmu \hbox {m}$ HCP, $270\,\upmu \hbox {m}$ BCC and $270\,\upmu \hbox {m}$ HCP idealised Cartesian soil geometries. Error bars indicate the standard deviation computed over ten computational replicates. The control hairs are ‘grown’ into an empty domain with no explicit soil geometry. The explicit hairs interact with 3D soil geometry

The explicit fluid-coincident hair length (Fig. 8c, d) is defined using the 3D hydrated textural phase representation from the SRXCT data. We consider hairs in this phase to have access to fluid over their entire area. To compare the results parameterised with explicit soil structure against the control results, approximations of ($L_{\mathrm{r},\mathrm{wet}}$) were computed from the controls, according to ($L_{\mathrm{r},\mathrm{wet}} =jL_{\mathrm{r},\mathrm{tot}}$), where *j* is the volume fraction of the non-primary mineral (i.e. hair-accessible) domain that is occupied by the hydrated textural phase (Fig. 7e, f). This simulates the assumption that fluid is homogenously distributed throughout the soil (as is the case in most bulk soil descriptions).

## Results

We now describe the results of the virtual hair growth studies in different geometries. The length profiles with distance from the root are taken as the key metric of interest. Fluid-coincident hair lengths are also compared between explicit and control cases, to determine whether explicitly considering the soil geometry changes the overall prediction and thus the proportion of hair length assumed to be active in P uptake.

### Experimental Determination of Hair and Soil Parameters

The mean hair lengths for the three biological replicates were $134\,\upmu \hbox {m}$ (SD = 45), $138\,\upmu \hbox {m}$ (SD = 55), and $151\,\upmu \hbox {m}$ (SD = 53), respectively. Fluid volume fractions for the entire syringe barrels, established via gravimetry, were 0.167, 0.241 and 0.231, respectively. The fluid volume fraction for the Cartesian soil sample was 0.250.

### Idealised Cartesian Scenarios

Figure 9 shows hair length profiles for idealised soil domains. Since no fluid distribution was inferred, only growth conditions $\hbox {A}_{1}\hbox {F}_{0}$ and $\hbox {A}_{1}\hbox {F}_{0}$ were considered. Packing arrangement had a greater influence on the profile of $(L_{\mathrm{r},\mathrm{tot}})$ than the soil particle size. In the case of the BCC packing, the $(L_{\mathrm{r},\mathrm{tot}}$) profiles for the explicit replicates were virtually identical to those of the control replicates. Both particle sizes (90 and $270\,\upmu \hbox {m}$) introduced a periodicity to the hair length profiles with distance from initiation, which followed the inter-centroid distance of the particles. This was more pronounced for $\hbox {A}_{1}\hbox {F}_{0}$ than for $\hbox {A}_{0}\hbox {F}_{0}$. In both geometries, the $\hbox {A}_{1}\hbox {F}_{0}$ condition yielded explicit hair length profiles that were higher proximal to the root surface than the controls. This effect was much more pronounced under condition $\hbox {A}_{1}\hbox {F}_{0}$ than condition $\hbox {A}_{0}\hbox {F}_{0}$ for both particle sizes and both packing geometries.

**Fig. 10:**
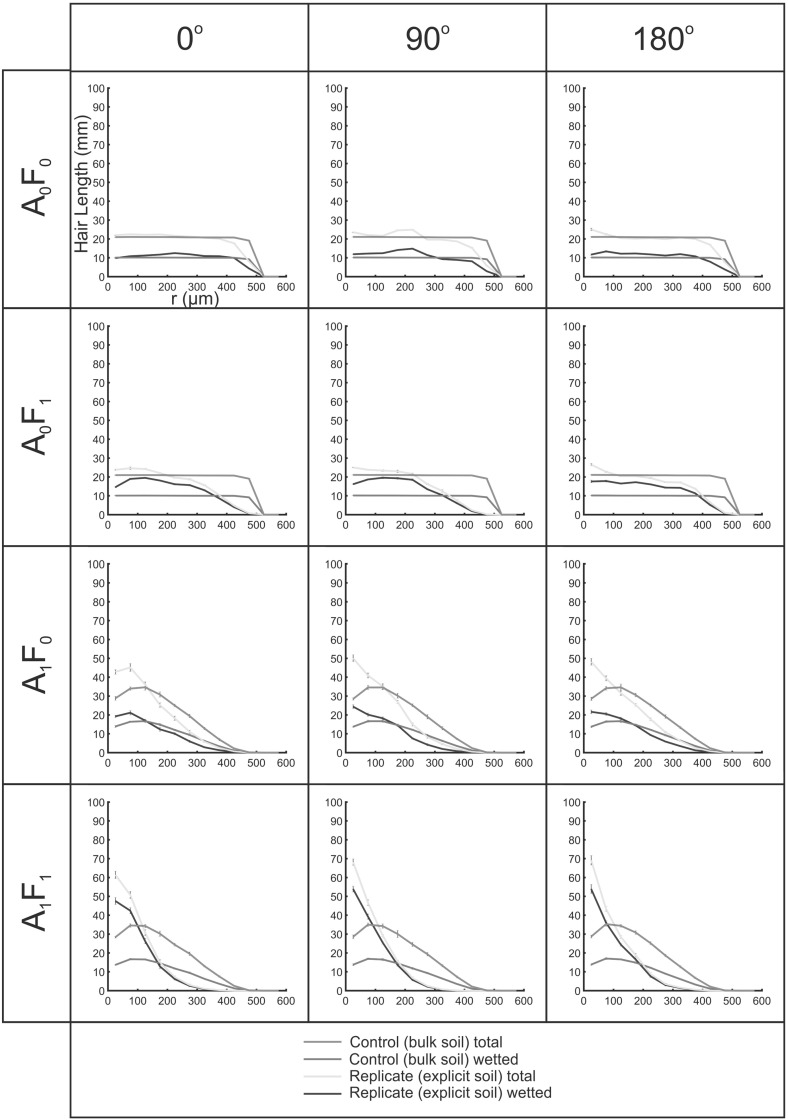
(Color figure online) Hair length density profiles with distance (*r*) from the initiating surface for hairs grown from three different faces into an SRXCT-derived Cartesian soil geometry. Error bars indicate the standard deviation computed over ten computational replicates. The control hairs are ‘grown’ into an empty domain with no explicit soil geometry, while the explicit hairs interact with 3D soil geometry

**Fig. 11:**
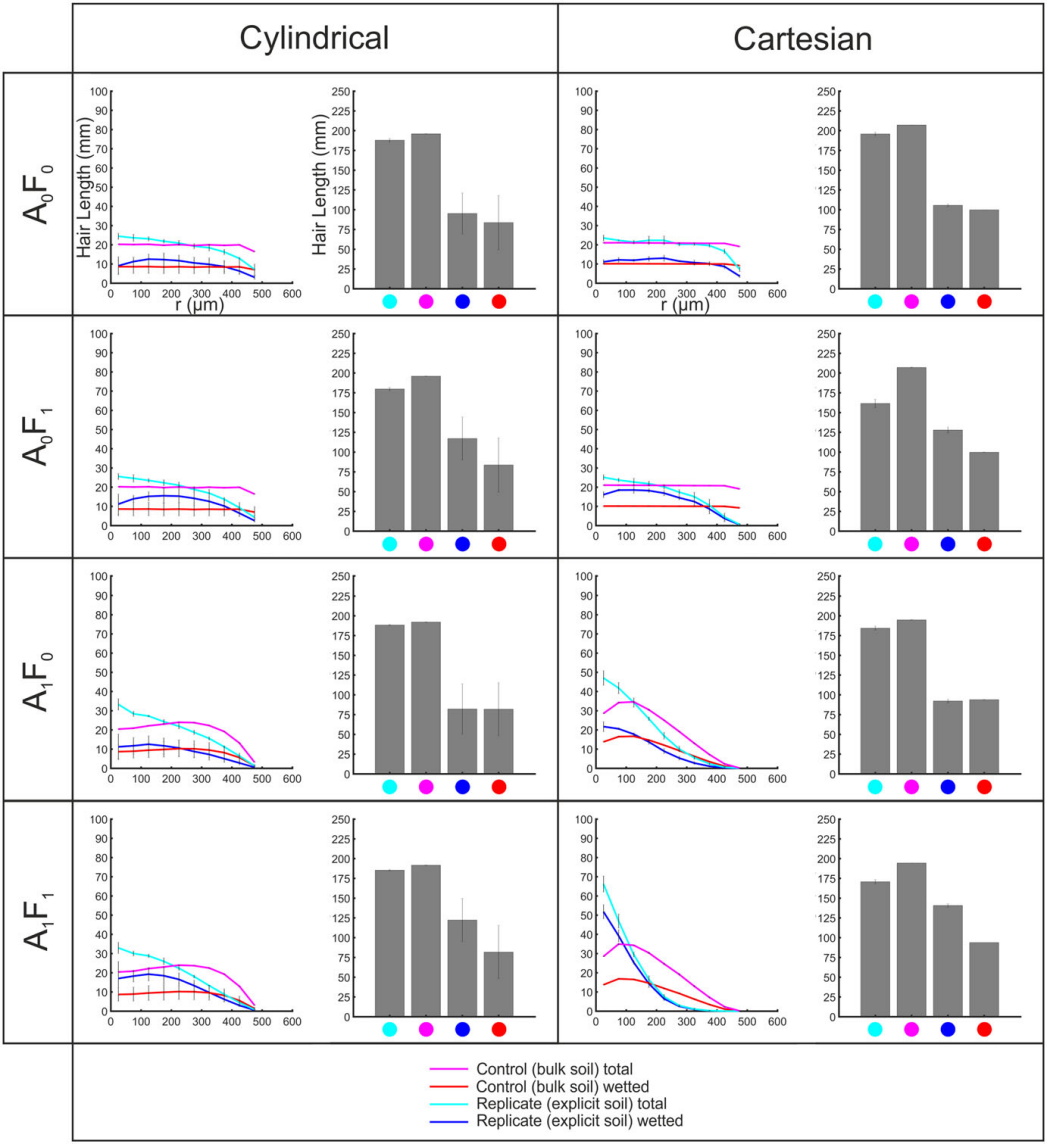
(Color figure online) Hair length density profiles with distance (*r*) from the initiating surface, and bar charts of total integrated hair length, for each growth condition, averaged over all cylindrical and Cartesian SRXCT geometries. Error bars show one standard deviation either side of the mean ($n = 3$)

### SRXCT-Derived Cartesian Scenarios

Figure 10 shows hair length profiles with distance from the root $(L_{\mathrm{r},\mathrm{tot}}$ and $L_{\mathrm{r},\mathrm{wet}})$ for growth conditions $\hbox {A}_{0}\hbox {F}_{0}$, $\hbox {A}_{1}\hbox {F}_{0}$, $\hbox {A}_{0}\hbox {F}_{1 }$ and $\hbox {A}_{1}\hbox {F}_{1}$ in the Cartesian case. Some heterogeneity of $L_{\mathrm{r},\mathrm{tot}}$ and $L_{\mathrm{r},\mathrm{wet}}$ between the different growth directions was evident. However, the same general trends were observed for each growth condition. For growth condition $\hbox {A}_{1}\hbox {F}_{0}$, the explicit fluid-coincident length profiles were similar to those of the respective control (bulk) replicates. Under all other conditions, the fluid-coincident length densities of explicit replicates were substantially elevated above those of the control (bulk) replicates in the near-root ($\sim 0$–$400\,\upmu \hbox {m}$) region. Under all growth conditions, the fluid-coincident hair length density profiles $(L_{\mathrm{r},\mathrm{wet}})$ were lower in magnitude than, but similar in characteristic shape to, the total hair length density profiles ($L_{\mathrm{r},\mathrm{tot}}$). A smaller offset between the two was observed under $\hbox {A}_{0}\hbox {F}_{0}$ and $\hbox {A}_{1}\hbox {F}_{0 }$ compared to $\hbox {A}_{0}\hbox {F}_{1 }$ and $\hbox {A}_{1}\hbox {F}_{1}$.

Figure 11 shows the total fluid-coincident length for the explicit computational replicates of growth conditions, relative to the homogenous approximations, averaged over the three directions. Considering the total integrated fluid-coincident lengths, the elevation of the explicit over the bulk results $\left( \frac{L_\mathrm{v,wet,explicit} }{L_\mathrm{v,wet,control} }\times 100\% \right)$ followed the order: $\hbox {A}_{1}\hbox {F}_{1 }(150\%)>\hbox {A}_{1}\hbox {F}_{0 }(128\%)>\hbox {A}_{0}\hbox {F}_{0 }(105\%) > \hbox {A}_{1}\hbox {F}_{0 }(98\%)$.

### SRXCT-Derived Cylindrical Scenarios

**Fig. 12:**
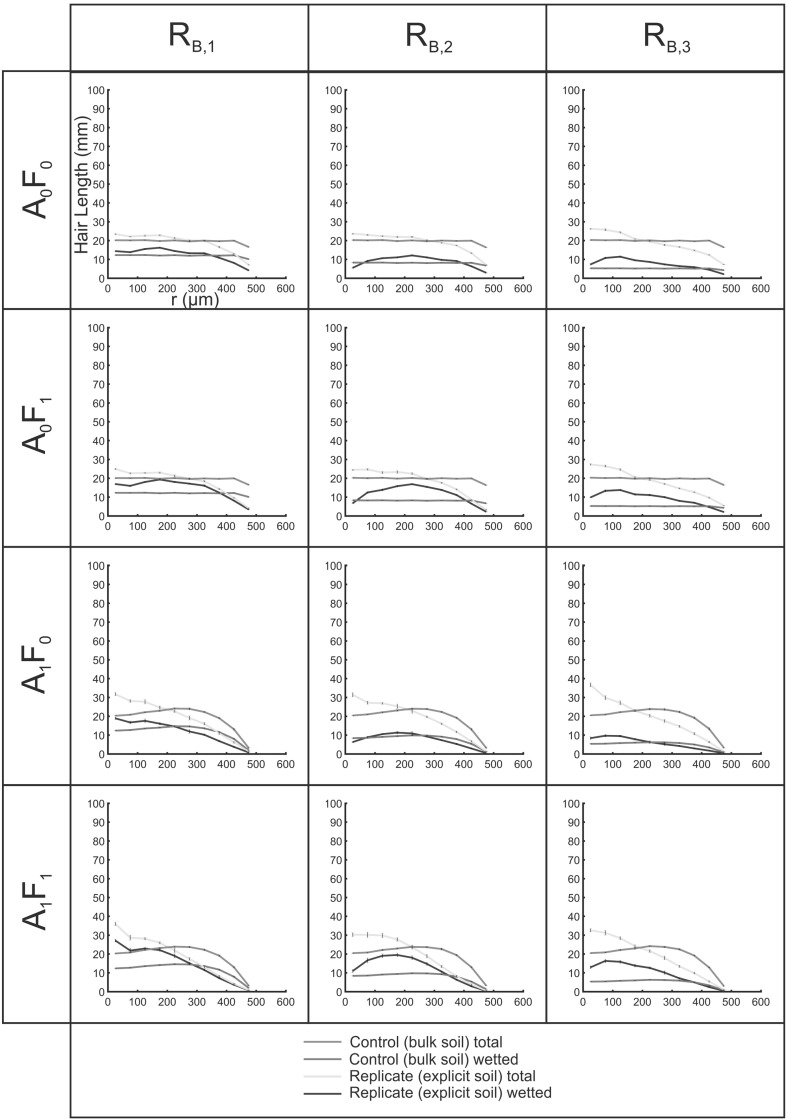
(Color figure online) Hair length density profiles with distance (*r*) from the initiating surface for hairs grown in three SRXCT-derived soil geometries, seeded from the epidermal surfaces of primary roots of *Oryza sativa cv. DJ123*. Error bars indicate the standard deviation computed over ten computational replicates. The control hairs are ‘grown’ into an empty domain with no explicit soil geometry; the explicit hairs interact with 3D soil geometry

Figure 12 shows the total and fluid-coincident hair length density profiles with distance from the root ($L_{\mathrm{r},\mathrm{tot}}$ and $L_{\mathrm{r},\mathrm{wet}}$) for growth conditions $\hbox {A}_{0}\hbox {F}_{0}$, $\hbox {A}_{1}\hbox {F}_{0}$, $\hbox {A}_{0}\hbox {F}_{1 }$ and $\hbox {A}_{1}\hbox {F}_{1}$. For all conditions, the explicit fluid-coincident length was higher than the control in the near-root region, with the profiles crossing each other at $\sim 100$–$200\,\upmu \hbox {m}$ from the root surface.

Fluid-coincident length density profiles followed the same trends as observed in Cartesian geometries, being significantly higher than the controls in all cases except under growth condition $\hbox {A}_{1}\hbox {F}_{0}$ where the profiles were similar. For the conditions in which hairs were constrained by fluid interactions (F$_{1}$), there was a marked elevation of fluid-coincident length density in the near-root region, sustained for $\sim 300$–$400\,\upmu \hbox {m}$ away from the root surface; the zone incorporating the entire range of experimentally measured hair lengths (Fig. 12).

Figure 11 shows the total fluid-coincident length for the explicit computational replicates of growth conditions, relative to the homogenous approximations, averaged over the three biological replicates. Considering the total integrated fluid-coincident lengths, the elevation of the explicit over the bulk results $\left( \frac{L_\mathrm{v,wet,explicit} }{L_\mathrm{v,wet,control} }\times 100\% \right)$ followed the order: $\hbox {A}_{1}\hbox {F}_{1 }(149\%)> \hbox {A}_{1}\hbox {F}_{0 }(140\%)> \hbox {A}_{0}\hbox {F}_{0 }(114\%) > \hbox {A}_{1}\hbox {F}_{0 }(100\%)$.

**Fig. 13:**
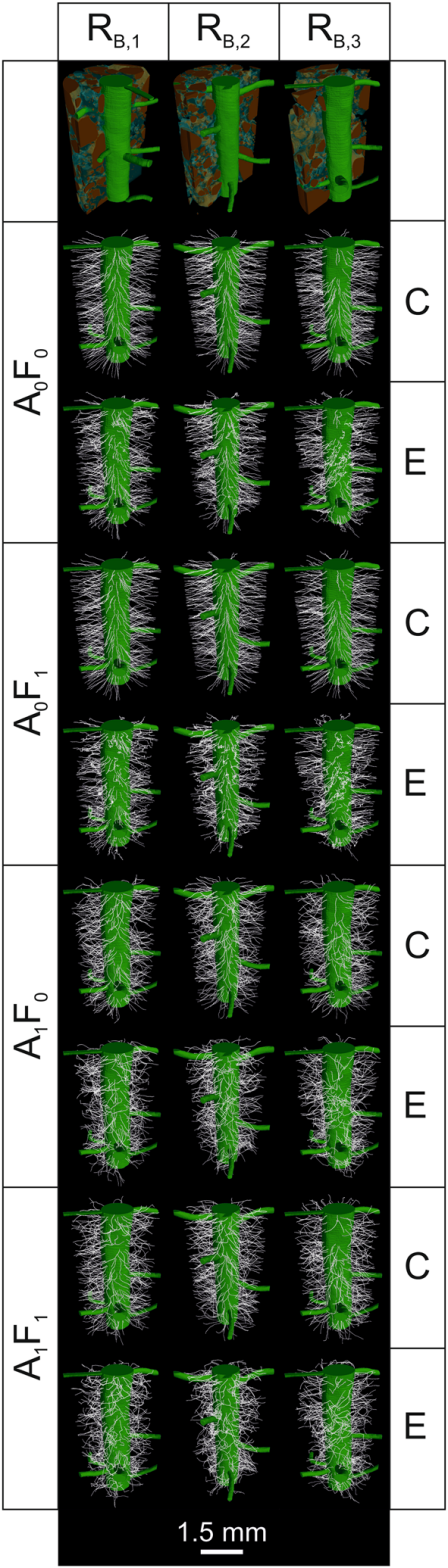
(Color figure online) 3D renderings of synthetic hair sets grown into the cylindrical rhizosphere soil geometries derived from SRXCT of three biological replicates. Morphology of explicit hairs (*E*) and control (bulk) hairs (*C*) is shown for each growth condition

Figure 13 shows 3D renderings of each of the biological replicates under each of the growth conditions. The tortuosity of hairs in explicit replicates (from greatest to least) followed the order $\hbox {A}_{1}\hbox {F}_{1}>\hbox {A}_{1}\hbox {F}_{0}>\hbox {A}_{0} \hbox {F}_{1}>\hbox {A}_{0}\hbox {F}_{0}$. Increased tortuosity in explicit versus control replicates was highly evident.

## Discussion

The use of SRXCT imaging and novel plant growth assay has allowed the development of a 3D rhizosphere-imaging protocol. Data acquired in this manner have recently been used to parameterise nutrient dynamic models (Keyes et al. 2013; Daly et al. 2016), but their potential for informing growth models has not until now been explored. Using a heuristic image classification approach, we converted SRXCT data to discretised 3D descriptions of rhizosphere geometry, enabling an explicit structural root hair growth model to be parameterised at the micron scale. This allowed the potential growth behaviour and morphology of root hairs in occluding phases to be explored *in silico*, using realistic rhizosphere geometry.

In both Cartesian (flat root surface) and cylindrical (curved root surface) geometries, the explicit fluid-coincident hair length profiles $(L_{\mathrm{r},\mathrm{wet}}$) in SRXCT geometries showed the most substantial deviation from the control (bulk) condition under conditions in which hair growth was constrained by surface energy effects at the boundaries of fluid-rich phases (F$_{1}$). Since P diffuses in soil water, located predominantly in the hydrated textural phase for soils at or below field capacity, mathematical modelling schemes may significantly underestimate uptake if they treat soil as a bulk and assume fluid is homogenously distributed. If hair growth is not strongly governed by these interactions with fluid-rich phases, then a bulk saturation-based approximation of the fluid-coincident length, as used in homogenous soil models (Ma et al. 2001; Leitner et al. 2010b), may represent an acceptable simplification, since explicit and control fluid-coincident hair lengths were closely matched in the F$_{0}$ cases (Fig. 11).

Comparison of Cartesian and cylindrical SRXCT geometries evidenced the same trends in the influence of different growth conditions upon both total fluid-coincident length and the profiles of fluid-coincident length with distance from the initiating surface $(\hbox {A}_{1}\hbox {F}_{1}>\hbox {A}_{0}\hbox {F}_{1}>\hbox {A}_{0} \hbox {F}_{0}>\hbox {A}_{1}\hbox {F}_{0})$. Since nutrient uptake modelling at the hair scale is generally concerned with relative rather than absolute magnitudes, this suggests that the use of the simpler Cartesian geometries is acceptable in these models (Leitner et al. 2010b; Roose and Zygalakis 2010; Zygalakis et al. 2011).

The hair growth algorithm presented here represents only one approach, the emphasis being on a formulation whose parameters were relatively straightforward to determine. With increased understanding of the constitutive physical properties of hairs and soils, more sophisticated mechanistic descriptions of their interactions may be feasible. For this study, governing assumptions were drawn from both qualitative observations of SRXCT data and canonical understanding of root hair development. Certain dynamic behaviours are difficult to verify, such as the degree to which pressure exerted by an extending hair tip can deform the soil matrix. Such information would allow more precise arguments to be made regarding how hairs and/or soil particles should deform during mechanical interaction.

The very complex morphological descriptions revealed by SRXCT raise significant issues with understanding the many interlinked biophysicochemical processes within this key zone (Roose et al. 2016). Even using the most sophisticated imaging and image processing techniques, the delineation of soil phases in this study still represents a substantial simplification of the true particle size distributions characteristic of rhizosphere soil. However, incorporating descriptions of real soil to parametrise models of putative hair behaviour enables the study of root hairs to move beyond the aeroponic, gel or cryo-sectioning approaches canonically used (Lauter et al. 1996; McCully 1999; Ma et al. 2001).

An obvious modification to the growth mesocosm is to incorporate a system for more accurate control and quantification of soil water content. In this study, the total gravimetric water content for each biological replicate was in excess of the hydrated textural phase volume fraction observed in the small region (0.6% of the syringe volume) imaged via SRXCT. The heterogeneity of the soil at the millimetre scale, and the varying water potential with tube height, make it possible that water content outside the field of view (particularly near the chamber base) was substantially elevated. Modification of the mesocosms to incorporate a tension apparatus for control of water potential (Quinton et al. 2009) represents an obvious refinement for future work, but at SRXCT scales, heterogeneity is likely to remain an issue.

Numerical simulations will be required to probe how different hair length profiles might influence nutrient depletion dynamics around the root. In the F$_{1}$ conditions, explicit replicates generated profiles of ($L_{\mathrm{r},\mathrm{wet}}$) that were markedly skewed towards the near-root region compared to the control (bulk) replicates. This was true for both Cartesian and cylindrical geometries. If fluid interactions do strongly influence growth, competition for P between hairs and the root may be greater than would be predicted using a homogenous (bulk) soil model. Much depends on both the localisation and mobility of strongly sorbed nutrients in the soil matrix, and the comparative P uptake capacity of root and hairs. In the few models which consider root hairs explicitly, it has been necessary to assume hair uptake potential is equal to that of the root due to a lack of hair specific data (Zygalakis et al. 2011). Models parameterised by soil and hair geometries derived from SRXCT and functional–structural models can in future be used to explore the influence of different hypotheses regarding spatiotemporally heterogenous uptake potential.

The few mathematical models that explicitly consider soil structure have idealised it as single-phase grains with either simple surface reactions or a dual porosity regime (Leitner et al. 2010b; Ptashnyk and Roose 2010; Zygalakis et al. 2011). Soil geometry from SRXCT suggests that a multi-phase soil model imposing dual porosity in the hydrated textural phase and zero-flux across primary mineral grain surfaces may be more accurate. Complementary experimental techniques to identify spatial distribution of nutrient sources and fluid films will further enhance the accuracy of rhizosphere-scale models. X-ray and spectroscopic data fusion (Hapca et al. 2011; Hu et al. 2014) and neutron tomography (Carminati et al. 2010) are promising techniques in this regard. We stand at an exciting crossroads in rhizosphere research which will require truly interdisciplinary collaboration.

## Conclusion

SRXCT imaging combined with suitable plant growth mesocosms can provide non-destructive, three-dimensional descriptions of rhizosphere morphology at very high resolution ($\sim 1\,\upmu \hbox {m}$). By using these data to directly parameterise the mechanical interactions of a functional–structural growth model, more sophisticated understanding of this highly complex domain was gained. The application of a novel structural root hair growth algorithm allowed 3D hair ideotypes to be computationally generated and allowed the testing of predictions regarding hair growth dynamics and development of new hypotheses regarding the uptake of sparingly soluble nutrients. The hair morphologies produced are of a format suitable for finite-element meshing for numerical solution of cell problems, which will in future allow mathematical homogenisation of rhizosphere-scale dynamic models, producing simpler models that can be applied in an image-based manner at the root system scale (Daly et al. 2017). The results of the morphometric studies conducted suggest that soil particle packing has more influential effect that particle diameter on hair morphology and that physical interactions between hairs and soil water are likely to be an important determinant of hair P uptake.

## Electronic supplementary material

Below is the link to the electronic supplementary material.

Supplementary material 1 (rtf 54334 KB)
