# Supplementary material for: An Explicit Structural Model of Root Hair and Soil Interactions Parameterised by Synchrotron X-ray Computed Tomography
Source: Bull Math Biol. 2017 Oct 13;79(12):2785–813. doi: 10.1007/s11538-017-0350-x (PMC5709508; doi:10.1007/s11538-017-0350-x)
Supplement: Supplementary file 1 — Supplementary material 1 (rtf 54334 KB) [file 11538_2017_350_MOESM1_ESM.rtf]

Supplementary Materials
1.	Standardisation of the plant growth assay
Briefly, a two-part seed cup was designed using a CAD package (SolidWorks 2013, Dassault Systèmes, SolidWorks Corp., Santa Monica, CA), the upper part of which contains soil and a germinated seed.  Seminal roots are guided by the cup morphology into seven 2.5 mm apertures.  The lower part mates with the base of the cup, extending the root guides into each of 7 syringe barrels (root chambers) that are lightly interference-fitted to the base.  The lower portion including the root chambers is housed in a foil-wrapped 50 ml centrifuge tube which occludes light during the growth period.  The seed cup parts were fabricated in ABS plastic using an UP! 3D printer (PP3DP, China).  Figure S1.1 shows a schematic of the assembled microcosm, with S1.2 showing a 3D render from an X-ray CT image of a rice plant (Oryza sativa cv Varyla) following 14 d growth in a Eutric Cambisol.  The guiding of roots into individual root chambers is evident. Preparation for imaging of individual barrels proceeds according to the following protocol: 1) a razor blade is passed between the upper and lower components of the seed cup, 2) the individual chambers are gently removed from the lower part, and each opening sealed with paraffin film to minimise evaporative losses during imaging.  The assay represents an inexpensive, reusable and repeatable approach to growing individual roots in the small soil volumes necessitated by the parameters of SRXCT imaging.  The barrels that contain roots are easily identified after separation of the seed cup components, being evident from the cut cross sections within the root guides. The use of transparent barrels allows regions where roots grow against the barrel walls to be avoided, ensuring that only roots entirely surrounded by soil are imaged.  Post-imaging washing of the sample allows the location of the imaged region relative to the root tip to be determined.    

2.	SRXCT imaging protocol
An electron accelerator (synchrotron) is used to generate a beam of highly coherent, monochromatic X-ray light highly suited for discriminating between the constituent phases of small (<10 mm) objects.  After passing through the sample, the attenuated X-ray beam is converted to visible light, magnified by an optical microscope objective, and projected onto a charge-coupled device (CCD) that acquires 2D digital images (radiographs) which are subsequently reconstructed computationally into 3D volumes (1).  Samples are mounted on a rotational stage, with 1601 projections being acquired during a continuous rotation of ð radians.  A double-crystal, multilayer monochromator allows the beam energy to be optimized for the sample, with the high beam coherence facilitating secondary contrast enhancement through exploitation of phase shift.  The exploitation of phase shift differentials between sample constituents is of particular benefit when imaging biological samples, characterized as they are by weak X-ray absorption and the closely-matched attenuation coefficients of constituent materials (2).  Adjustable edge enhancement can be simply achieved and tuned by a 1-dimensional shift of the detector away from the sample along the beam axis (3).  For this study, a modest edge enhancement was used so as not to over-emphasize mineral/gas interfaces and thereby introduce detrimental complexities to the digital image processing workflow.  Following radiograph acquisition, the computational reconstruction of dark and light corrected sinograms allows final computation of a 16-bit volume, usually as a stack of 2D .tiff files.  These data had an isometric spatial resolution of 1.6 ìm.  For further detail on SRXCT methods as applied to roots and soil, the reader is referred to a standard text (4). 


            


Supplementary Figure S1 –The growth microcosm used to image the intact rhizospheres of individual nodal roots. (1) Roots are guided from a seed chamber (yellow) into soil-filled 1 ml syringe barrels of ≈4 mm internal diameter. (2) A cutaway of an X-ray CT scan of the entire assembly demonstrates the guiding of rice roots (Oryza sativa cv Varyla at 12 d) into syringe barrels.


3.	Microstructural soil changes evident under soil drying
Figure S2 shows representative soil structures revealed using SRXCT imaging at 1.5 ìm resolution.  S2.1 shows a sample of Eutric Cambisol at a water content of ~20% by volume.  The soil macrostructure comprises primary mineral grains (with effective diameters at the 100-500 ìm scale) within a textural matrix that appears continuous and largely homogenous at the imaging resolution.  In Figure S1.1, a representative primary mineral grain and textural-phase region are highlighted in red and blue respectively.  Figure S2.2 shows a sample of the same soil following 24 h drying at 74oC  in a vacuum oven (Heraeus) that reduced the water content to <1% by volume.  No alteration of the primary mineral grain structure is evident, but the observable structure of the textural matrix was clearly significantly altered by the removal of soil water.  These changes correlate with those observed by others in resin-mounted thin sections, which found that the dominant drying-induced microstructural changes in finely-aggregated soils were pore enlargement and cracking within the textural phase (5).


Supplementary Figure S2 – Representative hydrated (≈25% b.v.) and dried (<1% b.v.) soil regions in Eutric Cambisol sieved for a particle size range of 1000-1680 ìm. (1) In the hydrated sample, the primary minerals (red) and hydrated textural phase (blue) that comprise the non-gaseous components of the soil matrix are distinct. (2) In the dried matrix the primary mineral phases are not qualitatively different.  However loss of water from the textural phase has resulted in an increased degree of visible porosity and micro-cracking.

4.	Heuristic soil classification 
The algorithm uses a fast random forest classifier (6) to assign image pixels to different material classes (in this case: 'gas', 'fluid/textural', and 'primary mineral') based on the outputs of an iterative training routine that incorporates user feedback.  Discrete sets of representative pixels defined for each material class (the 'training' datasets) are used to construct a classifier model.  A number of decision trees (a 'random forest') is constructed, each of which contains an implementation of the classifier model.  The forest then estimates the most likely class to which any given pixel in an image belongs, with each tree 'voting' independently and the overall result for the forest determining the class to which each pixel is allocated.  The data associated with each pixel (used to train and apply the classifier) comprise the outputs of a user-defined set of measures (such as the gradient computed using the Sobel operator), each of which is computed on a pixel-wise basis.  The classifier accuracy can be iteratively improved via the user checking the correlation of output regions and the raw data, adding further training pixels to refine the classifier behavior where necessary.  Once the congruence of the material classification and the raw data is acceptable, the material classes can be exported as a three-dimensional geometry.  

5.	Assessing sensitivity of growth algorithm to step length and angle
The growth algorithm operates according to a stepping routine, with a segment of length r being 'grown' each iteration at an angle of  satisfying , but always takes a value satisfying  unless this would entail growing into a disallowed region (i.e. soil primary mineral).  An obvious concern with such a routine is the sensitivity of the hair growth mechanics to changes in these parameters, and particularly the question, what is the influence of step length changes on grown hair morphology?  
We carried out a small study to assess the influence of changing step length on the hair morphology.  Single hairs were grown with identical demand length () of 400 units, but with step sizes of 1, 2, 5, 10, 20 and 50 respectively.  The angle constraints were the same in each instance, with k=10 sets () where the lower bound of  is  and the upper bound is .  A growth point will always be randomly selected from the non-zero set with the lowest value of k.  Thus large angular deviations (>) will only result if necessitated by collision with objects (in this case, either primary mineral grains or the domain boundary).
A single hair for each value of  was grown into a domain with rows of cylindrical pillars of diameter 5, 10, 15, 20, 25, 30, 35, 40, 45, 50, 55 and 60 units, aligned perpendicular to the initial direction of hair growth.  Figure S3 shows the resulting hair morphologies for Condition 1 (constant initiation vector) and Condition 2 (changing initiation vector).
It is observed that a step length of 1 produces undesirable behaviour, both in the overall length generated and in, the case of Condition 2, for the hair shape.  This is attributable to a break-down in the sphere method used to distribute the test points.  Because the hairs are generated using voxel data, the test sphere (with a radius equal to the step length) is superimposed onto a uniform grid, with the test points being assigned to the nearest node on the grid.  With a step length of 1, this means that test points are in fact all distributed across the vertices and face centroids of a square with edge length 2.  Thus only four angles are available for each growth step.  Under Condition 2, where the initiation vector is the preceding growth step, this produces the erroneous morphology seen in figure S3.  Under Condition 1, where the growth angle for each step is picked relative to the initial direction of growth, the morphology is not able to diverge to the same degree.
Though the length of the skeleton using a step length of 1 is the same as the demand length (the input length used to parameterise the growth model), the apparent length once the hair skeletons are dilated can be lower than the demand length.  This is clearly seen for step length 1 under Condition 1.  This is due to a 'staircasing' effect on the unit scale produced by limited growth angles that is smoothed out once the hairs are dilated. 
Larger step lengths produce hairs with smoother morphology relative to the hair scale.  The limiting factor in length is the requirement not to 'hop' over small particles.  For this reason, the step length (r) should be kept below the diameter of the smallest particles in the soil geometry.  Filtering out particles with volumes smaller than  ensures this does not occur.  The other issue would occur with interactions with features of very low thickness (membrane-like structures), but in practice the primary mineral grains features observed in the soil are almost always quasi-spherical.
The algorithm is most stable with step lengths in the range , with a value of 15 being the default.  With additional data on dynamics of hair growth or the constitutive properties of hairs in situ, this and other parameters could be refined.  For example, the stiffness of hairs will influence the degree of tortuosity during growth, a factor which could be implemented in the model by constraining or relaxing the angular constraint.  The relationship between step length and the spatial scale of features in the soil geometry will also influence hair morphology (Figure S3).     However the difficulty of determining constitutive properties and dynamics of hairs and soils at this scale means that assumptions have necessarily been made, such as assuming non-deformable soil (such that hairs rather than soil deform upon interaction), allowing per-step angular deviation of hairs up to , and setting the step size to be smaller than the smallest particle diameters in order to avoid hairs traversing primary phases.  Though these assumptions produce realistic-looking hair morphologies, future characterisation of soil and hair properties at the micron scale may enable these assumptions to be replaced by more accurate mechanistic descriptions. 


Supplementary Figure S3 – a sensitivity analysis was carried out with hairs of step lengths 1, 2, 5, 10, 20 and 50, in domains with obstacles of varying diameter.  Very small step lengths are seen to produce erroneous results relative to observed hair morphologies, in the form of highly tortuous hair paths and large angles of divergence, neither of which are characteristic of hair morphologies observed on gel media or in soil (either using cryo-SEM or SRXCT). however for both growth conditions, selection of step lengths in the range 10-50 produce hair-like morphologies that are not qualitatively highly divergent.

6.	Validating correlation of demand, sampled and virtually grown length distributions
It was important to validate that the distribution of grown lengths matches that of the sampled lengths, and that this in turn matched the original distribution used as an input to the hair growth workflow.  Correlation was tested using two normal distributions, D1 () and D2 ().  Weibull parameters were fitted to the distributions using MATLAB.  Next, 121 fully virtual lengths were picked from each distribution, being used to parameterize fully virtual hair growth from a planar surface of dimensions 1 mm by 1 mm.  Hairs were grown in both the absence and presence of an idealized sphere of diameter 500 ìm, centered at 1000 ìm from the growth plane.  Figure S4 shows the hair paths, along with the correlation of length distributions, verifying that in impeded and unimpeded cases, and at different distributive spreads, the distribution of grown hairs matches very closely the original distribution.


Supplementary Figure S4 – Correlation of base, picked and grown hair length distributions is demonstrated for two different hair length distributions D1 () and D2 (), in the presence and absence of an obstacle (sphere with diameter D=500 ìm). (1-3) D1, no obstacle, (4-6) D1, obstacle, (7-9) D2, no obstacle, (10-12) D2, obstacle.


Supplementary Figure S5 – Histograms of hair lengths measured from the root region within the FOV of the SRXCT scan for biological replicates Rb,1, Rb,2 and Rb,3.


7.	References
1. 	Stampanoni M, Groso A, Isenegger A, Mikuljan G, Chen Q, Bertrand A, et al. Trends in synchrotron-based tomographic imaging: the SLS experience. Proc. SPIE; 2006. p. 63180M – 63180M. 
2. 	Weitkamp T, Diaz A, David C, Pfeiffer F, Stampanoni M, Cloetens P, et al. X-ray phase imaging with a grating interferometer. Opt Express. 2005;13:6296–304. 
3. 	Pagot E, Fiedler S, Cloetens P, Bravin A, Coan P, Fezzaa K, et al. Quantitative comparison between two phase contrast techniques: diffraction enhanced imaging and phase propagation imaging. Phys Med Biol. 2005;50:709–24. 
4. 	Anderson SH, Hopmans JW. Chapter 3. Soil-water-root Processes: Advances in Tomography and Imaging. Soil Science Society of America; 2013. p. 39–68. 
5. 	Bresson LM, Moran CJ. Micromorphological study of slumping in a hardsetting seedbed under various wetting conditions. Geoderma. 2004;118:277–88. 
6. 	Breiman L. Random forests. Mach Learn. Springer; 2001;45(1):5–32. 
